# Micro-Magnetic and Microstructural Characterization of Wear Progress on Case-Hardened 16MnCr5 Gear Wheels †

**DOI:** 10.3390/ma11112290

**Published:** 2018-11-15

**Authors:** Marina Knyazeva, Julian Rozo Vasquez, Leonard Gondecki, Max Weibring, Fabian Pöhl, Monika Kipp, Peter Tenberge, Werner Theisen, Frank Walther, Dirk Biermann

**Affiliations:** 1 Department of Materials Test Engineering, TU Dortmund University, 44227 Dortmund, Germany; julian.rozo@tu-dortmund.de; 2 Chair of Industrial and Automotive Drivetrains, Ruhr University Bochum, 44780 Bochum, Germany; leonard.gondecki@rub.de (L.G.); max.weibring@rub.de (M.W.); peter.tenberge@rub.de (P.T.); 3 Chair of Materials Technology, Ruhr University Bochum, 44780 Bochum, Germany; poehl@wtech.rub.de (F.P.); theisen@wtech.rub.de (W.T.); 4 Institute of Machining Technology, TU Dortmund University, 44227 Dortmund, Germany; kipp@isf.de (M.K.); biermann@isf.de (D.B.)

**Keywords:** wear, non-destructive testing, micro-magnetic testing, surface fatigue

## Abstract

The evaluation of wear progress of gear tooth flanks made of 16MnCr5 was performed using non-destructive micro-magnetic testing, specifically Barkhausen noise (BN) and incremental permeability (IP). Based on the physical interaction of the microstructure with the magnetic field, the micro-magnetic characterization allowed the analysis of changes of microstructure caused by wear, including phase transformation and development of residual stresses. Due to wide parameter variation and application of bandpass filter frequencies of micro-magnetic signals, it was possible to indicate and separate the main damage mechanisms considering the wear development. It could be shown that the maximum amplitude of BN correlates directly with the profile form deviation and increases with the progress of wear. Surface investigations via optical and scanning electron microscopy indicated strong surface fatigue wear with micro-pitting and micro-cracks, evident in cross-section after 3 × 10^5^ cycles. The result of fatigue on the surface layer was the decrease of residual compression stresses, which was indicated by means of coercivity by BN-analysis. The different topographies of the surfaces, characterized via confocal white light microscopy, were also reflected in maximum BN-amplitude. Using complementary microscopic characterization in the cross-section, a strong correlation between micro-magnetic parameters and microstructure was confirmed and wear progress was characterized in dependence of depth under the wear surface. The phase transformation of retained austenite into martensite according to wear development, measured by means of X-ray diffraction (XRD) and electron backscatter diffraction (EBSD) was also detected by micro-magnetic testing by IP-analysis.

## 1. Introduction

The lifetime of gears is limited by different damage mechanisms such as tooth root break, pitting or scuffing. Focusing on tooth flank fatigue, it is important to be able to monitor the fatigue progress over time and to understand the interlinking processes of initiation and growth of cracks as well as the interaction between the lubricant and the tooth material. Micro-pitting describes a wear and fatigue phenomenon, which mainly occurs on highly loaded and case-hardened tooth flanks in slow running gear stages of industrial gears. The fatigue phenomenon is decisively influenced by the geometry of the gear, the loading and the chemical reactions between the tooth material and the additives of the oil within the atmosphere [1]. The term micro-pitting describes the matt grey areas on tooth flanks, which are primarily formed on areas with negative sliding speeds and high sliding paths. The phenomenon starts first with short cracks on the tooth’s flank surface whereas the crack density increases with the number of load changes. At the same time, existing cracks grow deeper into the material or return back to the surface. At a certain crack density, small microscopic particles break out of the surface and the characteristic grey surface is formed. With an increasing number of microscopic outbreaks, a profile form deviation emerges, which starts at the tooth root.

The structural health monitoring (SHM) involves the development of non-destructive methodologies to measure and control the integrity of components at every moment [2]. In this work, the micro-magnetic testing is applied as a non-destructive technique to characterize the micro-pitting-related wear on the tooth flanks of gears, since its progress leads to microstructural changes correlated to the magnetic properties of the material. The results are compared with other techniques like profilometry; X-ray diffraction; and light and scanning electron microscopy, including electron backscatter diffraction analysis.

## 2. Materials and Methods

With the aim of the development of SHM-techniques, the focus of the present work is a multi-technique characterization of the flanks of gear teeth subjected to different states of wear, identified from 0 to 4, depending on different loading conditions. The investigation was performed beginning with characterization of wear progress on the surface, using a group of non-destructive techniques as profilometry via confocal white light interferometry, X-ray diffraction (XRD) and micro-magnetic testing, followed by a microstructural characterization via light and scanning electron microscopy (SEM). The micro-magnetic testing was applied for a depth-sensitive description of the properties of ferromagnetic materials and verified by means of the electron backscatter diffraction (EBSD) analysis, carried out on the cross-sections.

### 2.1. Wear Tests

In order to generate specimens from tooth flanks in different wear states, a specific test program based on the micro-pitting test according to FVA (Forschungsvereinigung Antriebstechnik) information sheet 54/7 [3] was performed on a standard FZG (Forschungsstelle für Zahnräder und Getriebebau) gear test rig [4]. This test method was defined to primarily analyze the influence of the lubricant on the formation of fatigue-related wear on the tooth flank.

**Figure 1:**
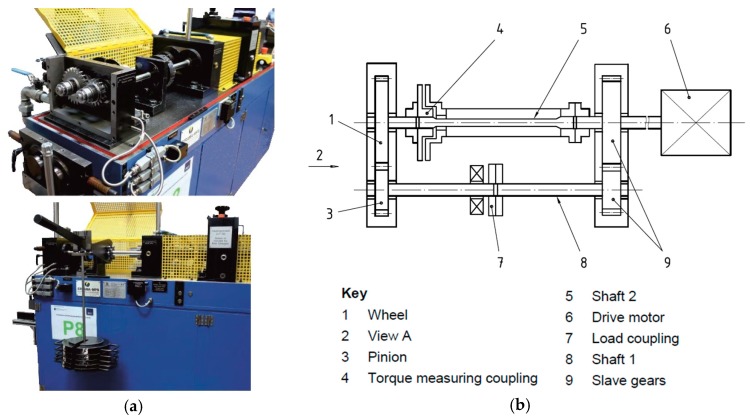
FZG gear test rig: (**a**) photos; (**b**) schematic drawings.

The operating conditions including the loading, the circumferential speed and the macro and micro geometry of the gear set were specified in a way that the contact between the surfaces of the tooth is under mixed friction conditions. The standard FZG gear test rig with a center distance of 91.5 mm consists of a test gear box containing the test gears and a strain gear box connected by couplings, as can be seen in Figure 1. A constant torque was applied to the system by tensioning the two halves of the load coupling at standstill. The test oil was injected into the tooth contact at a temperature of 90 °C with a flow rate of 2 l/min [5]. The gear set is made of 16MnCr5 (1.7131, SAE 5115) and the tooth geometry complies with the standard-C according to FVA 54/7 [3].

The test sequence defines four different load stages as outlined in Table 1. The first stage includes a short running-in at a pinion torque of 70 Nm and a second run with a pinion torque of 265 Nm. Both runs consist of 1.5 × 10^5^ load cycles. Stage two adds another 12 × 10^5^, stage three 30 × 10^5^ and stage four 45 × 10^5^ load cycles at a constant pinion torque of 265 Nm and a constant pinion speed of 2250 RPM. This test sequence was performed on two gear sets (1 and 2) whereas the test was always stopped after another load stage. Four sets of tooth flanks in different wear states (1–4) were generated. A synthetic industrial gear oil (ISO VG 100) was used throughout the entire test sequence.

### 2.2. Wear Characterization Methodologies

**Figure 2:**
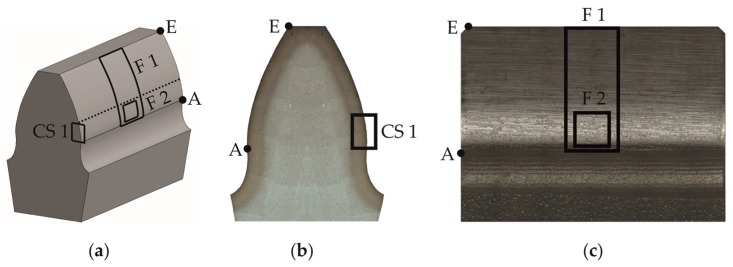
Areas of interest: (**a**) Three-dimensional scheme; (**b**) Cross-section; (**c**) Front view of the flank. CS: Cross-section; F: Flank; A: Used tooth root diameter; E: Tooth tip.

The areas of interest of micro-magnetic testing, microscopic analysis and micro-structural characterization of gears are shown in Figure 2.

#### 2.2.1. Characterization of Wear Progress on the Surface

**Figure 3:**
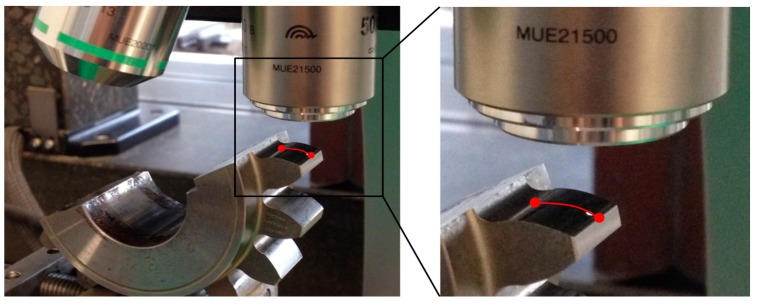
Tooth flank surface documentation of the pinion with an autofocus measuring device.

In order to determine the fatigue-related wear on the tooth flank, the profile form deviation of the gears was determined by a Klingelnberg PNC65 CNC-controlled gear measuring machine (Klingelnberg AG, Zurich, Switzerland). The profile form deviation is defined as the difference between the worn flank and its initial state. The area of interest F1 is given in Figure 2. An autofocus measuring device, by Alicona Imaging GmbH (Raaba/Graz, Austria) is used to generate a microscopic image of the tooth flank, as is presented in Figure 3, in order to document the temporal development of the surface of the tooth flank.

A topographic characterization on the tooth flank was performed using a confocal white light microscope NanoFocus µSurf (NanoFocus AG, Oberhausen, Germany). The area of this inspection is defined in Figure 2 as F1 and it reaches from the tooth root (point A in Figure 2) to the tooth tip (point E in Figure 2).

#### 2.2.2. Microstructural Characterization by Light Microscope and Scanning Electron Microscope

A microstructural characterization of the wear progress of the teeth flank surface was performed by means of light microscopy and scanning electron microscopy (SEM). The inspection was focused on the area of the flank close to the tooth root, specified in Figure 2 as F2, using the light microscope Zeiss Axio Imager (Carl Zeiss AG, Oberkochen, Germany) and the SEM Tescan Mira 3 XMU (TESCAN GmbH, Dortmund, Germany). A microstructural characterization of the cross-section area, defined as CS1 in Figure 2, was carried out in order to describe the wear progress on the surface and under it, in order to link these results with the micro-magnetic parameters.

The EBSD was performed over the cross-section, specifically on the area marked as CS1 of the Figure 2, using the SEM Tescan Mira 3 XMU, equipped with a DigiView EBSD camera and the Team™ software of Edax Inc., (Weiterstadt, Germany). The OIM analysis™ software was used for the post-processing of the results. The specimens undergo metallographical preparation including cutting out, grinding and polishing using conventional suspensions (6, 3 and 1 µm). At a final step, the surface was polished with a colloidal SiO_2_ suspension (oxide polishing suspension, OPS). The EBSD measurements were performed with the table tilted at 70°, using an electron beam energy of 25 to 30 kV, working distance circa 20 mm and magnification of 2500×, with a final inspection area of 20 µm × 20 µm.

#### 2.2.3. X-ray Diffraction (XRD)

Residual stresses and the amount of retained austenite were measured by means of X-ray diffraction (XRD) using a portable X-ray device type µ-X360 of Pulstec Industrial Co., Ltd. (Nakagawa, Japan). The measurements were performed on the area of interest F2 from Figure 2. Based on the cosα-method, introduced by Taira et al. [6] and further developed by Sasaki and Hirose [7], the device contains an image plate acting as a two-dimensional detector that enables the capturing of the entire Debye-ring at once during a single measurement. Further methodological details are available in References [8,9].

The measurements were conducted using CrKα-radiation with a wavelength of 2.29 Å, an X-ray tube voltage of 30 kV and a current of 1 mA, focused by a Φ = 0.5 mm collimator. Macroscopically determined elastic constants were given as E = 210 GPa and ν = 0.3. The lattice parameter was set to be 2.8664 Å for all samples. In consideration of the target material, the diffraction line h, k, l (211), the corresponding 2ϴ-diffraction angle of 156.3° and an x-ray incidence angle of ψ_0_ = 35° were chosen during the determination of residual stresses, which was adjusted to ψ_0_ = 18° for the measurement of retained austenite. The samples were positioned at a distance of 20 ± 1 mm, determined by means of the automatically measured 2ϴ-angle, for the measurement of retained austenite and the identification of residual stresses. 

The relative amount of retained austenite was calculated based on the area ratio of the detected austenitic peak profile, with regards to the total amount of both martensitic and austenitic share according to the detected peak intensities, and the corresponding FWHM-values (full width at half maximum), detected at half peak-maximum, such as the cosα-angle.

#### 2.2.4. Micro-Magnetic Testing

The micro-magnetic tests, based on the cyclic magnetization of a ferromagnetic material, were carried out on the area of interest F2, shown in Figure 2. The applied magnetic field strength H, the magnetic flux density B, also known as magnetic induction, and the magnetization M which is the vector sum of the magnetic moments of all the atoms of the material in a small volume, are related to each other with the magnetic constant or permeability of free space μ_0_, through Equation (1) [10].

(1) $$B=\mu_{0}\left(H+M\right),$$

**Figure 4:**
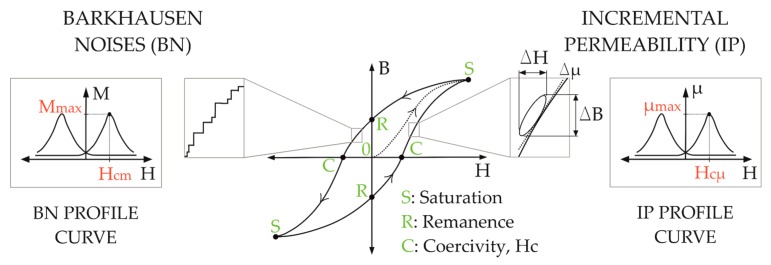
Scheme of hysteresis loop with main parameters of micro-magnetic analysis.

The magnetization curves obtained with the measuring equipment, describe the variation of the magnetic flux density B or the magnetization M, with respect to the applied magnetic field strength H. These curves were taken from the first quadrant of the hysteresis loop, as presented in Figure 4. From a demagnetized state in B = H = 0, the magnetization increases until a saturation point S. At this point, the magnetization is no longer reversible, and therefore, if the magnetic field is decreased, the magnetization will come back not along the initial line, but describing a new one along S-R. In the point R, at H = 0, the magnetic flux density B reaches a point called remanence or residual magnetization. A further increase of the magnetization will place the magnetization flux density B at a zero value, where point C can be found, known as the coercive force or coercivity H_c_. A further decrease of magnetization leads the curve to the negative part of the loop, reaching a saturation point and completing the hysteresis loop without crossing the initial null state [11].

Ferromagnetic materials are divided into different magnetic domains, which are spontaneously magnetized regions separated by domain walls. The magnetization vectors in these regions are oriented in different directions, but their sum is zero for the whole material, and the leakage of magnetic flux into the surrounding air space is reduced [12]. During the magnetization, the change of the flux density B proceeds by rotation of the magnetization and by the motion of the domain walls, which can find obstacles during the increment of the magnetic field strength H. After breaking these obstacles away, sudden jumps occur, called Barkhausen jumps. These discontinuous changes of B, induce voltage peaks that can be detected as Barkhausen noise, shown in the Figure 4, and these are used as one measuring strategy during the present study. Another important methodology is the incremental permeability Δμ, also illustrated in Figure 4, obtained by the ratio of the flux density swing ΔB to the corresponding field strength excursion ΔH [10].

The micro-magnetic testing was carried out by means of two devices. The 3MA II, manufactured by Fraunhofer IZPF (Saarbrücken, Germany), allows the analysis of harmonics (HA), Barkhausen noise (BN), incremental permeability (IP) and eddy current signals (EC). In BN analysis, the detected signals enable the creation of the BN profile curve, illustrated in Figure 4, showing the BN amplitude as a function of the magnetic field H, namely M(H). From this curve, the obtained parameters were the maximum amplitude M_max_ and the magnetization amplitude H at the point, which is the coercivity H_c_, reported in BN analysis as H_cm_. On the other hand, the IP curve, presented also in Figure 4, resulting from the superposition of two alternating magnetic fields, with differences in frequency and amplitude of magnetization (f_Field1_ >> f_Field2_ and H_Field1_ << H_Field2_). In this way, small hysteresis loops are created, whose slopes represent the incremental permeability Δμ and their projection over the magnetic field strength H, creates the profile curve µ(H), as presented in Figure 4. The coercivity H_c_ will be the magnetization amplitude H at the maximum permeability, μ. This is reported as H_cµ_ and is the coercive magnetic field acquired by IP analysis [13].

The other applied equipment was the FracDim manufactured by Fraunhofer IKTS (Dresden, Germany), suitable for the analysis of BN and harmonics, with the particularity of allowing the changing of filter bandpass, in order to determine the microstructure-sensitive magnetization in dependence of depth. With this device the maximum amplitude of the BN profile curve M_max_ was obtained for different ranges of bandpass frequencies. FracDim does not work directly with the magnetic field H, but with a correlated parameter called magnetic flow, Phi. Therefore, the BN envelope, M (Phi) will exhibit the maximum amplitude M_max_ at Phi_cm_, which is a parameter directly correlated with the coercive magnetic field H_c_ [14].

To assess the properties at different depths, the micro-magnetic tests were carried out using different ranges of bandpass frequencies. The electromagnetic signals of certain frequency pass through the electrically conducting material and are attenuated by eddy current damping, in a way that the amplitude of the field at a skin depth, δ, decays to 1/e (around 37%) of its value at the surface [15]. With this concept, it is possible to compute the skin depth effect using Equation (2), which has been widely used in many works [16,17].

(2) $$\delta =\sqrt{\frac{\rho }{\pi \;f\;\mu }},$$

where δ is the skin depth in mm, ρ is the electrical resistivity in Ω·mm^2^/m, f is the filter bandpass frequency in Hz, and μ is the magnetic permeability in V·s/A·m.

## 3. Results

### 3.1. Wear Progress on the Surface of Gear Wheels Teeth

**Figure 5:**
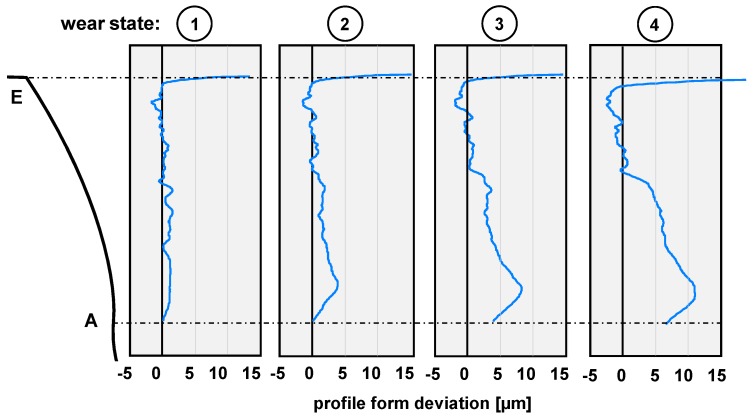
Profile form deviation of the gear teeth for the different wear conditions.

The following results were obtained from the non-destructive and destructive tests performed on the gear teeth, between the points A and E along the flank F, as defined in Figure 2. In Figure 5, the results of the profile form deviation measurements are given for a single tooth of each gear flank. In the wear state 2, a first local material removal starting at the tooth root with a depth of 4 μm appears. The maximum amount of wear continuously increases to 12 μm in wear state 4. Additionally, the local wear also grows to the pitch point.

**Figure 6:**
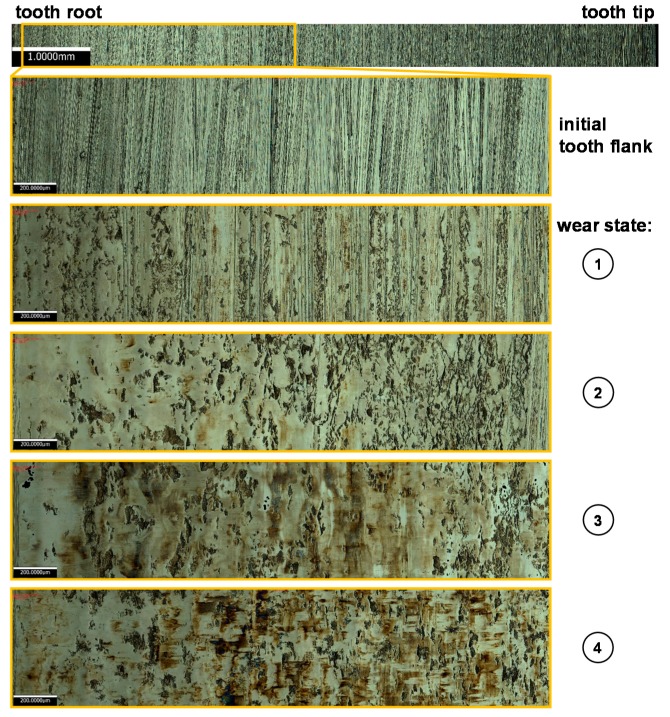
Surface development of gear teeth for the different wear conditions.

Figure 6 shows the microscopic images of tooth surfaces in all different wear states taken on inspection areas of the gear teeth defined in Figure 2 as F1. In the initial state, the grinding grooves and their orientation vertically to the circumferential rolling and sliding direction are clearly visible. With an increasing number of load cycles, the surface characteristics change significantly. Starting at the tooth root, the surface is smoothed towards the pitch point. Some microscopic break-outs on the tooth flank are visible but they are smeared up by the surrounding material.

**Figure 7:**
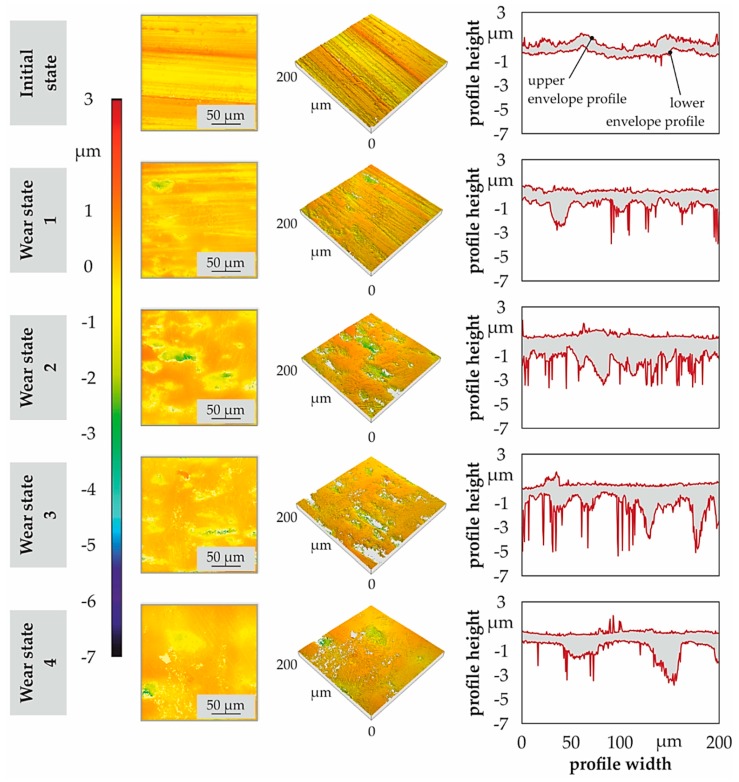
Surface topography of the gear teeth for the different wear conditions.

Figure 7 shows the surface topography of exemplary areas of 200 µm × 200 µm of the teeth, taken in the area F1 of Figure 2, for the initial ground surface and the different wear states. The upper and lower envelope profiles of surface topography describe the wear progress in dependence of loading states. By means of upper envelope profile, the flattening of the grinding marks with the formation of running tread surface is clearly recognizable. The simultaneous process of occurrence of micro-pitting is easy to distinguish on the lower envelope profile.

### 3.2. Microstructural Characterization

**Figure 8:**
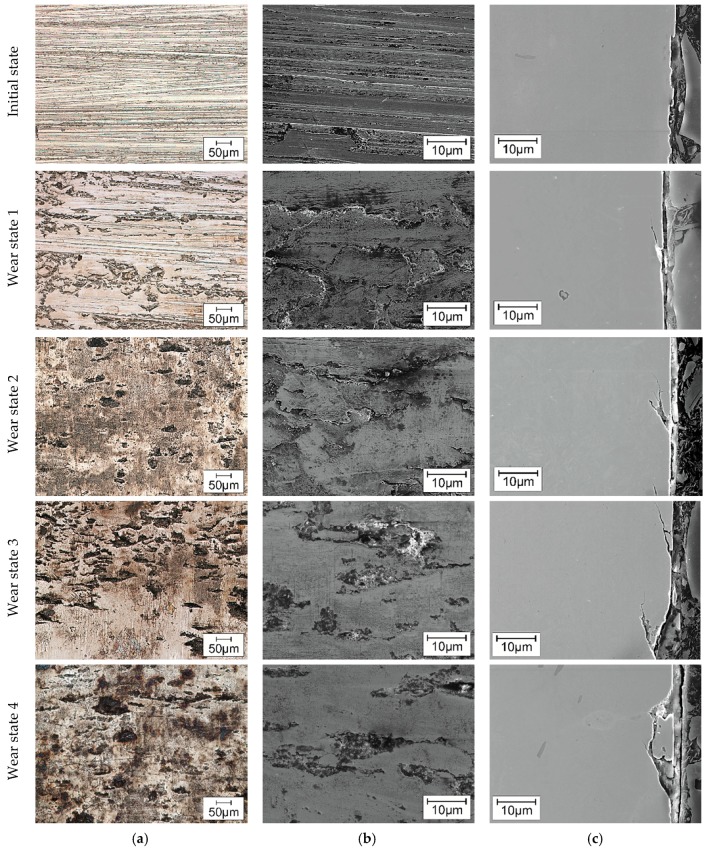
Microstructural characterization of wear in gear teeth by means of: (**a**) light microscope (200×) in area F2; (**b**) SEM on the surface (2000×) in area F2; (**c**) SEM on cross-section (2000×) in area CS1.

The results of microscopy obtained by means of light microscope and scanning electron microscope are presented in Figure 8. 

According to the specified areas of interest CS1 and F2 in Figure 2, the surface inspection was performed on the direct contact zone of the flanks, close to the tooth root. The evolution of the surface in each wear condition is consistent with the results presented in Figure 6. The initial state of the gear wheels teeth surface shows the grinding marks of the fabrication process. As it was revealed by means of confocal microscopy, after a short loading time in wear condition 1, many of the grinding marks disappeared and the surface became flatter forming running treads. Some 2–3 µm-deep cracks are already recognizable in the cross-section at this wear state. In the following wear conditions, simultaneously to the formation of treads, spalls of micro-pitting, recognizable as grey areas, appear, and their density and size grow with the wear progress. With an increase of loading cycles on the teeth, the enforced apparition and increment of size and depth of cracks is recognizable. For instance, in the wear condition 2, the crack size increases up to ca. 6 µm and reaches in the two final wear states approx. 8 µm. The material which was broken due to surface fatigue was shredded and smeared up in the spalls, which prevents an additional abrasion and slows down the wear progress at later wear states.

### 3.3. Electron Backscatter Diffraction

**Figure 9:**
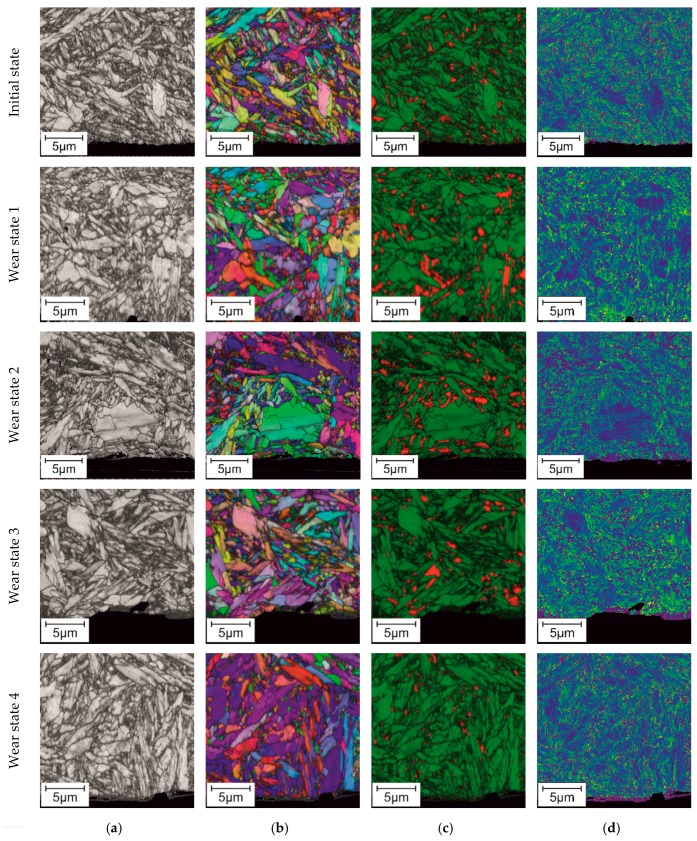
EBSD on cross-sections of gear teeth (surface is shown at the bottom): (**a**) Fit map; (**b**) Inverse Pole Figure (IPF) map; (**c**) Phase map: green: bcc-lattice (martensite), red: fcc-lattice (austenite); (**d**) Kernel Average Misorientation (KAM) respect the first neighbor: blue: minimum misorientation, red: maximum misorientation.

The results of the electron backscatter diffraction (EBSD) measurements representing an integral analysis of the microstructure up to 20 µm under the surface are summarized in Figure 9.

**Figure 10:**
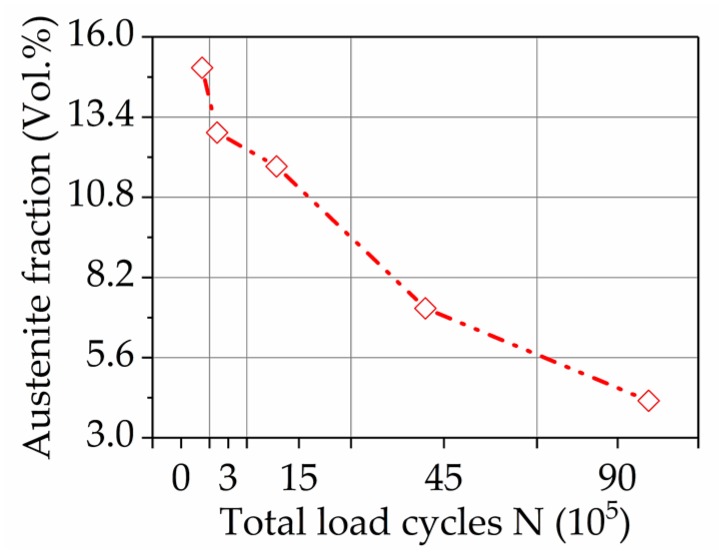
Austenite fraction of different wear conditions of gear teeth obtained by EBSD.

The dark areas in Fit-maps (Figure 9a) indicate the distorted crystal lattice, and therefore, the grain boundaries and areas with high deformation become well recognizable. Due to its strongly distorted bcc-lattice, the former retained austenite transformed into martensite can be indicated by dark areas in Fit-maps. The Inverse Pole Figure (IPF)-map (Figure 9b) is a representation of the different orientations of the crystal lattice, and the Phase-map (Figure 9c) indicates the phases; therefore, it is possible to characterize the austenite amount and its transformation in dependence of wear progress. The Phase-map reveals also the quantitative transformation of retained austenite into martensite, given as a graph in Figure 10. The Kernel Average Misorientation (KAM) (Figure 9d) quantifies the average misorientation around the measurement points of the maps, with respect to the first nearest neighbour. As expected, bigger misorientations are present at the grain boundaries equivalent to the information presented in Fit-maps. No correlation of wear progress and increase of deformation were detected.

### 3.4. X-ray Diffraction

**Figure 11:**
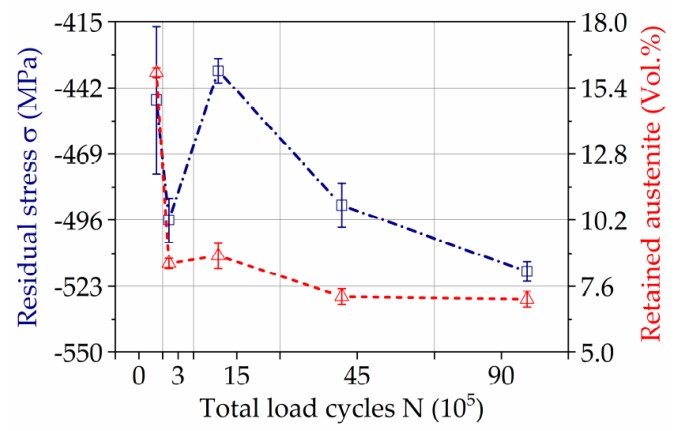
Residual stresses and amount of retained austenite, measured by x-ray diffraction analysis for the different wear conditions.

As illustrated in Figure 11 and summarized in Table 2, the amount of retained austenite is the highest for the initial condition and considerably lower for all conditions after wear testing.

However, the amount of retained austenite is predominantly decreasing with increasing amounts of wear, as it drops from an initial amount of 16% down to 7% for wear condition 4. The residual stress analysis revealed that the analyzed positions have negative residual stresses in all conditions. Generally, the compressive stresses tend to further decrease from condition 0 to 4 (from −447 MPa to −517 MPa).

### 3.5. Micro-Magnetic Testing

**Figure 12:**
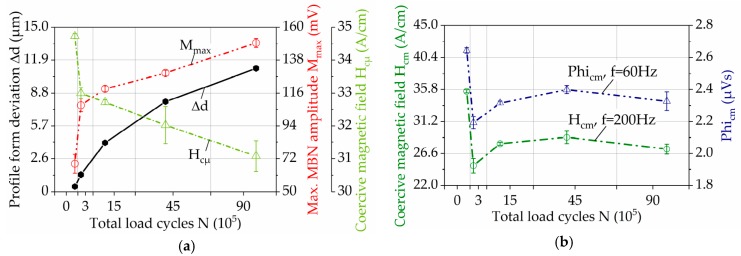
Results of micro-magnetic testing: (**a**) M_max_ and H_cµ_ by BN and IP, respectively, using 3MAII; (**b**) H_cm_ and Phi_cm_ by BN-analysis using 3MAII and FracDim, respectively.

The results of the micro-magnetic investigation are reported in Figure 12. In Figure 12a, the maximum amplitudes of the Barkhausen noise curve, M_max_ and the coercive magnetic force, and H_cµ_, obtained by the incremental permeability profile, are shown. These two parameters are compared with the progress of the profile form deviation Δd. The deviations reported in Figure 12 correspond to the average of measurements on three gears teeth, performed according to Figure 5. It can be seen that the value of M_max_ increases proportionally to wear progress, shown in the profile deviation. Inversely, the coercivity parameter H_cµ_ presents a linear decrease with the augment of the wear.

Another result of the micro-magnetic investigation is that the coercive magnetic force obtained by the Barkhausen noise profile curve H_cm_, correlates with the parameter Phi_cm_. The development of these parameters, presented in Figure 12b, is phenomenollogically similar to changes of residual stresses and retained austenite, obtained by XRD analysis (Figure 11). 

**Figure 13:**
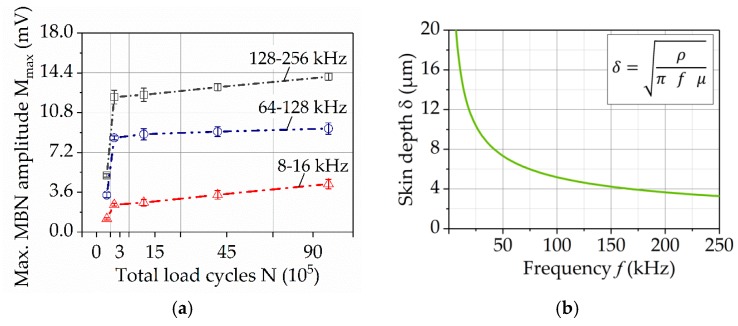
(**a**) Maximum amplitude of the BN profile curve for different filter bandpass frequencies, using FracDim; (**b**) Correlation of bandpass frequencies and depth, calculated based on the equation for skin effect.

Using different filter bandpass frequencies, the results of M_max_ were obtained and reported in Figure 13a for the different wear states. The behaviour of M_max_ for each filter bandpass frequency is consistent with the results presented in Figure 12a, presenting an incremental development for higher loading cycles. In each individual wear state, it can be seen that increased M_max_ values are registered for higher ranges of bandpass frequencies, which demonstrates that the highest wear state is on the surface and it diminishes when the depth increases.

To establish the correlation between skin depth and the filter bandpass frequency, Equation (2) was applied. For the material of the gear teeth, 16Mn5Cr, the electrical resistivity given in data sheets, is 0.16 Ω·mm^2^/m. The magnetic permeability was measured using Incremental Permeability, since this property is not constant but changes with the increase of the magnetization amplitude. Therefore the magnetic permeability was measured at a magnetization amplitude of 50 A/cm, for each wear state and calculated with 0.019 V·s/A·m as an average permeability. Based on this estimation, the approximated correlation of depth range, depending on the filter bandpass frequency, was calculated. The resulting curve given in Figure 13b illustrates that the higher the filter bandpass frequency, the closer to the surface is the analysis.

## 4. Discussion

### 4.1. Surface Investigation

As the contact situation was chosen to be under severe mixed friction conditions, the material removal starting at the tooth root and propagating towards the pitch point is to be expected, as illustrated in Figure 5. The mechanisms leading to the wear-related fatigue are well publicized in References [18,19,20]. As the matching tip edge of the driven gear gets in contact with the driving pinion, the local Hertzian pressure exceeds the nominal pressure at the pitch point of 1.5 GPa and the gliding speed is at its negative maximum. Therefore, the induced shear stresses at the tooth surface initiate first cracks at the asperities of the grinding grooves which propagate with a length of a few µm against the direction of the thrust in the depth of the material. Under further loading, the cracks crosslink and single micrometer-sized wear particles break off (micro-pitting) at stochastically distributed points from the affected surface area. This continuous process leads to a material removal of the weakened tooth flank areas. Nevertheless, the microscopic images in Figure 6 underline the presence of a certain additive package in the base oil. Under the test conditions, a mineral base oil with no additives (e.g., anti-wear, extreme pressure) leads to a tooth surface almost entirely affected with micro pitting and a bigger local material removal. However, the used synthetic industrial gear oil almost prevents the formation of microscopic break outs as only a few are visible on the surface in wear state 4, as can be seen in Figure 6 [21].

### 4.2. Microstructural Characterization

The microscopic analysis by means of light and scanning electron microscope, presented in Figure 8, describe the evolution of wear progress on the contact surface. The initial state is the characteristic surface finishing for these components after manufacturing, where some striations can be noticed along the grinding direction. No cracks were indicated in cross-sections, but the surface imperfections are susceptible areas for crack initiation after 3 × 10^5^ cycles of load. On the subsequent wear states, due to the strain gradient during the different load conditions, so-called “frosted” areas appear, as well as the micro-spalling associated with the pitting, according to Reference [22]. The size and amount of spalls increase under higher load cycles, as well as the size and amount of cracks. These results are consistent with the profile form deviation (Figure 5) and the topography (Figure 7) investigations of the surfaces. The changes in the profile geometry can be explained and evidenced from a microscopical point of view as a sum of the before described defects over the whole surface.

The amount of retained austenite was measured by means of both X-ray diffraction (Figure 11) and EBSD-analysis (Figure 9). Due to the high carbon content after heat treatment (including case hardening) the microstructure consists of tempered martensite with approximately 16 % retained austenite. During wear testing the initial content of retained austenite is reduced to approximately 3–7%. This indicates a mechanically-induced transformation of retained austenite to martensite by cyclic stresses and induced strains during wear testing. As it was shown due to the combination of X-ray and EBSD techniques, the transformation of austenite to martensite occurs in a first step in the upper layer close to the surface. The martensitic transformation in underlying layers follows stepwise by a continuous increase of loading cycles. No severe plastic deformation in neighbor grains was detected by EBSD in underlying areas, as reported in KAM-maps result (Figure 9d), indicating the prevalence of stress-induced mechanisms of martensitic transformation. The transformation from austenite to martensite leads to an increase in volume, which results in the rise of compression stress and might have a positive effect on the wear behavior. In References [23,24], it was shown that retained austenite can have a beneficial effect on wear and fatigue behavior of steel and cast iron. However, the effect of retained austenite on the wear behavior is controversial and depends on the actual tribological system [25].

As mentioned above, the cyclic loading also leads to a rearrangement of residual stresses in the material. After heat treatment (quenching with martensitic phase transformation) the analyzed surface region (few µm depth) has high compressive stresses that tends to further decrease during cyclic loading. The initial residual stress state can highly influence the deformation behavior since there is a superimposition with the contact stresses during wear testing. Generally, the further reduction of residual stresses can be attributed to accumulation of plastic strain, transformation of retained austenite and damage processes. In general, residual stresses can also reduce and suppress crack initiation and propagation.

### 4.3. Micro-Magnetic Testing

The microstructural investigations with the aim to evaluate the micro-magnetic analysis show good correlation between parameters such as the maximum amplitude of the Barkhausen noise curve M_max_, significantly increasing by the wear progress and corresponding to the profile deviation Δd, as well as the arising and growing of cracks. Simultaneously, the trend of coercive magnetic field H_cµ_, acquired by incremental permeability, presents a linear decrease, since M_max_ and the coercive field force H_c_ are opposed to each other [13]. The evolution of M_max_ and H_cµ_ helps to estimate the degree of deformation and surface damage done to the wear, since the microstructural changes affect the response of the magnetic domains. These results agree with previous works, where the micro-magnetic parameter M_max_, increases linearly with the raise of the engineering stress [26,27,28,29]. A sudden jump in both micro-magnetic parameters M_max_ and H_cµ_ occurs after 3 × 10^5^ cycles, mainly caused by phase transformation from austenite to martensite, formation of cracks and increased dislocation density. Due to the fact that surface fatigue is the dominating wear mechanism in the present experimental setup, an increase of defect density and lattice distortion occurs mainly in a thin layer close to the surface, as it can be evaluated in the Fit-maps acquired by means of EBSD. The fraction of dark areas in Fit-maps significantly expands by increasing the loading cycles, mostly due to austenite transformation.

The coercive magnetic fields H_cm_ and Phi_cm_ acquired by Barkhausen noise analysis (Figure 12b) show high sensitivity to the development of the residual stresses and correlates with the amount of retained austenite (Figure 11). The sudden drop of the coercive magnetic field after 3·× 10^5^ cycles follows the general trend of coercivity presented in Figure 12a, mainly caused by cracks formation and austenite transformation. By following wear conditions, a further decrease of the retained austenite and of the compressive residual stresses occurs, leading, according to References [26,27,28,29], to increasing of the coercive field force H_cm_ and Phi_cm_. The further decrease of both parameters indicates the strong concurrent influence of crack formation, as it was detected by coercive field force H_cµ_.

One of the most important possibilities of the micro-magnetic testing is the depth-dependent determination of microstructural changes under the surface. According to the concept of the skin depth effect [15], the magnetic signal decays at a determined distance, depending on the frequency, the permeability and conductivity of the material. This correlation is presented in the trend curves of M_max_ in Figure 13a. There, the M_max_ increases with the wear progress in all analyzed depths. The higher bandpass filter frequency representing the depth close to the surface shows the highest M_max_ values, which is in accordance with the fact that the plastic deformation and phase transformation manifest at first on the surface. The characteristic jump between the initial state and the first wear condition is also most distinctive for the trend curve representing areas close to the surface. Both facts indicate the concentration of deformation and damage in this area, associated with martensitic transformation and the appearance of cracks, as it was verified by means of XRD and EBSD-analysis. The surface cracks indicate surface fatigue as a prevailing wear mechanism. 

It can be concluded that the wear degree decreases gradually with the depth, presenting a maximum deformation and damage on the surface and can be successfully analyzed via micro-magnetic measurements. 

## 5. Conclusions

The combination of different techniques for surface analysis like profilometry, topography analysis, X-ray diffraction, microscopy and micro-magnetic testing allows for the acquiring of comprehensive information of the superficial state of the gear wheels tooth flanks. The results reveal quantitative information regarding the numerical deviation of the geometries in order to validate the wear progress. The qualitative assessments of wear development on the surface with respect to the formation of running surface, origin and growing of defects like micro-pitting and cracks help to identify wear mechanisms as surface fatigue.

The micro-magnetic testing was successfully applied for a non-destructive characterization of the wear progress on the surface of flanks of gear teeth. The results were verified with conventional techniques for microstructural characterization. On one hand, the micro-magnetic parameters such as the maximum amplitude of the Barkhausen noise curve and the coercivity analyzed by incremental permeability are more sensitive to the wear progress, due to their correlation with the profile deviation. On the other hand, the coercivity acquired by Barkhausen noise analysis shows high sensitivity with the behaviour of the austenite transformation and the evolution of residual stresses, obtained by means of X-ray diffraction. A depth-sensitive analysis performed by means of micro-magnetic testing, using different filter bandpass frequencies during magnetization shows good correlation with profile of residual stress under wear surface. Therefore, the micro-magnetic measurements were successfully verified as a tool for comprehensive analysis of wear progress and can be applied for Structure Health Monitoring.

## Figures and Tables

**Table 1:** Test sequence for each tooth flank [5].

Gear Set	Flank	Wear State	Loading			
			Pinion Torque (Nm)	Hertzian Contact Stress at Pitch Point (MPa)	Load Cycles (1 × 10^5^)	Total Load Cycles (1 × 10^5^)
1	A	1	70	795	1.5	3
			265	1547	1.5	
	B	2	70	795	1.5	15
			265	1547	1.5	
			265	1547	12	
2	A	3	70	795	1.5	45
			265	1547	1.5	
			265	1547	12	
			265	1547	30	
	B	4	70	795	1.5	90
			265	1547	1.5	
			265	1547	12	
			265	1547	30	
			265	1547	45	

**Table 2:** Residual stresses and amount of retained austenite, measured by x-ray diffraction analysis for the different wear conditions.

Wear Condition	Stress, σ (MPa)	Retained Austenite (Vol. %)
0	−447 ± 30	16.0 ± 0.2
1	−496 ± 9	8.5 ± 0.2
2	−435 ± 5	8.8 ± 0.5
3	−490 ± 9	7.2 ± 0.3
4	−517 ± 4	7.1 ± 0.3
